# Supplementary material for: Complicated Streptococcus agalactiae Sepsis with/without Meningitis in Young Infants and Newborns: The Clinical and Molecular Characteristics and Outcomes
Source: Microorganisms. 2021 Oct 3;9(10):2094. doi: 10.3390/microorganisms9102094 (PMC8540989; doi:10.3390/microorganisms9102094)
Supplement: Supplementary file 1 [file microorganisms-09-02094-s001.zip › microorganisms-1384247-supplementary.pdf]

Supplemental Table S1. Relationships between sequence type and serotype in 188 invasive GBS isolates in CGMH, 2003-2020

Sequence type	Serotype							
	Ia (%)	Ib (%)	II (%)	III (%)	IV (%)	V (%)	VI (%)	Total (%)
ST1	1 (3.2)	-	4 (100)	-	-	4 (57.1)	5 (100)	14 (7.4)
ST12	-	13 (92.9)	-	-	-	1 (14.3)	-	14 (7.4)
ST17	-	-	-	113 (89.7)	-	-	-	113 (60.1)
ST19	-	-	-	10 (7.9)	-	-	-	10 (5.3)
ST23	15 (48.4)	-	-	-	-	1 (14.3)	-	16 (8.5)
ST24	9 (29.0)	-	-	-	-	-	-	9 (4.8)
ST268	2 (6.5)	-	-	-	-	-	-	2 (1.1)
ST890	3 (9.7)	-	-	1 (0.8)	-	1 (14.3)	-	5 (2.7)
Others	1 (3.2)	1 (7.1)	-	2 (1.6)	1 (100)	-	-	5 (2.7)
Total (%)	31 (16.5)	14 (7.4)	4 (2.1)	126 (67.0)	1 (0.5)	7 (3.7)	5 (2.7)	188 (100)

ST: sequence type
